# Usage and Dose Response of a Mobile Acceptance and Commitment Therapy App: Secondary Analysis of the Intervention Arm of a Randomized Controlled Trial

**DOI:** 10.2196/mhealth.5241

**Published:** 2016-07-28

**Authors:** Elina Mattila, Raimo Lappalainen, Pasi Välkkynen, Essi Sairanen, Päivi Lappalainen, Leila Karhunen, Katri Peuhkuri, Riitta Korpela, Marjukka Kolehmainen, Miikka Ermes

**Affiliations:** ^1^ VTT Technical Research Centre of Finland Ltd Tampere Finland; ^2^ Department of Psychology University of Jyväskylä Jyväskylä Finland; ^3^ Vincit Oy Tampere Finland; ^4^ Institute of Public Health and Clinical Nutrition University of Eastern Finland Kuopio Finland; ^5^ Institute of Clinical Medicine, Clinical Nutrition Kuopio University Hospital Kuopio Finland; ^6^ Faculty of Medicine, Pharmacology, Medical Nutrition Physiology University of Helsinki Helsinki Finland; ^7^ VTT Technical Research Centre of Finland Ltd Kuopio Finland

**Keywords:** mobile apps, Acceptance and Commitment Therapy, retrospective study, adherence

## Abstract

**Background:**

Mobile phone apps offer a promising medium to deliver psychological interventions. A mobile app based on Acceptance and Commitment Therapy (ACT) was developed and studied in a randomized controlled trial (RCT).

**Objective:**

To study usage metrics of a mobile ACT intervention and dose-response relationship between usage and improvement in psychological flexibility.

**Methods:**

An RCT was conducted to investigate the effectiveness of different lifestyle interventions for overweight people with psychological stress. This paper presents a secondary analysis of the group that received an 8-week mobile ACT intervention. Most of the analyzed 74 participants were female (n=64, 86%). Their median age was 49.6 (interquartile range, IQR 45.4-55.3) years and their mean level of psychological flexibility, measured with the Acceptance and Action Questionnaire II, was 20.4 (95% confidence interval 18.3-22.5). Several usage metrics describing the intensity of use, usage of content, and ways of use were calculated. Linear regression analyses were performed to study the dose-response relationship between usage and the change in psychological flexibility and to identify the usage metrics with strongest association with improvement. Binary logistic regression analyses were further used to assess the role of usage metrics between those who showed improvement in psychological flexibility and those who did not. In addition, associations between usage and baseline participant characteristics were studied.

**Results:**

The median number of usage sessions was 21 (IQR 11.8-35), the number of usage days was 15 (IQR 9.0-24), and the number of usage weeks was 7.0 (IQR 4.0-8.0). The participants used the mobile app for a median duration of 4.7 (IQR 3.2-7.2) hours and performed a median of 63 (IQR 46-98) exercises. There was a dose-response relationship between usage and the change in psychological flexibility. The strongest associations with psychological flexibility (results adjusted with gender, age, and baseline psychological variables) were found for lower usage of *Self as context* related exercises (B=0.22, *P*=.001) and higher intensity of use, described by the number of usage sessions (B=−0.10, *P*=.01), usage days (B=−0.17, *P*=.008), and usage weeks (B=−0.73, *P*=.02), the number of exercises performed (B=−0.02, *P*=.03), and the total duration of use (B=−0.30, *P*=.04). Also, higher usage of *Acceptance* related exercises (B=−0.18, *P*=.04)

was associated with improvement. Active usage was associated with female gender, older age, and not owning a smart mobile phone before the study.

**Conclusions:**

The results indicated that active usage of a mobile ACT intervention was associated with improved psychological flexibility. Usage metrics describing intensity of use as well as two metrics related to the usage of content were found to be most strongly associated with improvement.

**Trial Registration:**

ClinicalTrials.gov NCT01738256; https://clinicaltrials.gov/ct2/show/NCT01738256 (Archived by WebCite at http://www.webcitation.org/6iTePjPLL)

## Introduction

Digital health interventions hold a promise of providing help and support more continuously and cost-effectively than traditional face-to-face therapies. Mobile technologies, especially, enable seamless integration of interventions into the daily lives of users by partitioning the intervention content into smaller doses. Despite growing evidence of effectiveness of digital interventions, it is still unclear how the usage of interventions should be measured, how usage is associated with benefits, and how much interventions should be used in order to gain health benefits.

Several studies have found that active usage mediates the effects of interventions, in both face-to-face [1] and digital interventions [2-6]. Active digital intervention usage has been associated with outcomes in several areas of well-being and health, including weight loss [2,3], physical activity [4], mental well-being [5], and depression and anxiety [6].

The definitions and metrics used for describing usage vary between studies. Many studies describe usage in terms of adherence, which has been adopted from health care and means the extent to which a person’s behavior follows instructions or recommendations from a health care provider [7]. Christensen et al [8] defined adherence as “the extent to which individuals experience the content of the Internet intervention.” In many studies, adherence is defined in terms of expected intensity of usage, predefined by intervention designers. As found by Kelders et al [9], the most typical intended usage frequency in Internet interventions is once a week. However, when dealing with digital interventions, it may be difficult to predefine the measure of adherence as the required intensity may not be known. Therefore, it is important to collect and analyze in-depth data on usage in order to understand which metrics of usage are most strongly associated with effectiveness. The best metrics may also be different for different types of interventions. In their review, Donkin et al [10] found that the number of log-ins and the number of modules completed were the most commonly reported measures of usage. They also found that in interventions targeting aspects of physical health, the number of log-ins was most consistently associated with outcomes, whereas in psychological health interventions, module completion was the strongest predictor of success [10]. In their own study, Donkin et al [11] investigated the associations of several usage metrics and outcomes in a Web-based intervention for depression. They found that the total number of activities completed, the total number of minutes spent in the program, the average number of activities completed per log-in, and the average number of minutes online per log-in were associated with improvements [11]. They hypothesized that sometimes the inability to find dose-response relationships may be due to the metrics used [11].

Usage and outcomes of interventions are influenced by several factors that need to be taken into account when designing and studying interventions. For example, study design, interaction with a counselor, more frequent intended usage, and use of persuasive features have an impact on usage [9,12]. Most research indicates that the treatment outcomes in Internet-based treatments are associated with the amount of support—eg, [13,14]. In addition, adherence and retention can be improved by offering personal support (face-to-face, telephone, or email) before and during Internet interventions [15,16]. The background characteristics of users, such as sociodemographic factors, psychological traits, and prior experiences with technologies, may also influence usage. For example, in the review by Christensen et al [8], a lower level of baseline depression, younger age, and poorer knowledge of psychological treatments were associated with a higher adherence to depression interventions. Also, for generalized anxiety disorder, lower levels of symptoms were associated with higher usage. A trial on posttraumatic stress disorder found that women, older persons, those who lived with a partner, and those less experienced with a computer used the intervention more actively [8]. In a Web and mobile technology–assisted employee health promotion program, older age and poorer aerobic fitness were found to be associated with sustained usage of the technologies [17]. In the field of Internet interventions, there are some indications that treatments work best for slightly older people and those who are able to take responsibility for their treatment [18]. Having fewer comorbid depressive symptoms, a stable economic situation and an employment, and being in a relationship have also been found to predict positive outcomes [18].

Acceptance and Commitment Therapy (ACT) is a third-generation cognitive and behavioral therapy that aims to increase psychological flexibility. Psychological flexibility is the ability to fully contact the present moment, and to change or persist in behavior when doing so serves valued ends [19]. In other words, psychological flexibility consists of skills for handling one’s emotional reactions and thoughts in a constructive way as well as skills for promoting well-being through effective actions. Psychological flexibility has been found to be associated with better mental health and to predict future mental health [19]. It has also been associated with behavioral effectiveness, for example, job performance [20]. Psychological flexibility is established through 6 core processes, namely, *Being present*, *Self as context*, *Acceptance*, *Cognitive defusion*, *Values*, and *Committed action*, which are addressed in ACT interventions [19]. The processes are interrelated and overlapping, and therefore investigating their individual effects on well-being is challenging. However, preliminary evidence supporting the impact of *Acceptance* and *Cognitive defusion* processes exists [19].

There is already evidence on the effectiveness of ACT-based digital interventions. Several Internet interventions, especially, have shown promising results [21-27]. Bricker et al [23] evaluated a 3-month ACT-based smoking cessation intervention and found that the intervention achieved a quit rate of 23% (13/57) compared with 10% (6/58) in the control condition and that the participants logged in to the service, on average, 9 times and spent, on average, 19 minutes online per log-in. Carlbring et al [24] found that their 8-week ACT-based depression intervention resulted in large effects on depression, and the average number of modules completed was 5.1 out of 7 and the median log-in time was about 3.5 hours. Levin et al [26] evaluated a 3-week program for preventing mental health problems among college students and found improvements in depressive symptoms, and they reported that the majority of participants 70/76 (92%) completed both lessons and spent an average of 82 minutes in the program.

Mobile apps based on ACT and related techniques such as mindfulness are also emerging [28-32]. Bricker et al [28] converted their Web-based smoking cessation intervention into a self-paced mobile intervention. In an 8-week study, a quit rate of 13% (10/80) was achieved for the ACT intervention compared with 8% (7/84) for the control app, and the participants self-reported opening the app, on average, 37 times [28]. Heffner et al [33] further investigated the usage of the smoking cessation app and the associations between feature usage and quitting. They were able to identify several features that significantly predicted smoking abstinence and found that only 2 of the 10 most actively used features predicted smoking abstinence, that is, the users used less effective features more actively. Ly et al [29] developed an ACT-based self-help program combining a mobile phone app and Web-based psychoeducation and found an effect size of 0.50 for psychological flexibility. The participants self-reported using the app a couple of times a week during the 4-week intervention [29]. In another study, Ly et al [32] evaluated an ACT-based mobile app for stress management and found a within-group effect size of 0.62 for perceived stress. They defined adherence as the minimum of 2 registered activities per week, and by this criterion, 16/36 (44%) participants adhered to the program for all of the 6 weeks of the intervention [32].

The aims of this study were (1) to investigate the dose-response relationship between usage and the change in psychological flexibility, (2) to identify the usage metrics that were most strongly associated with improvement in psychological flexibility, and (3) to study the associations between usage and baseline participant characteristics.

## Methods

### Overview

A randomized controlled trial (RCT; trial registration: ClinicalTrials.gov NCT01738256) was organized to study the effectiveness of 3 low-intensity lifestyle interventions against a no-intervention control. The interventions were (1) a face-to-face, group-based ACT intervention, (2) an ACT-based mobile intervention, and (3) a Web-based educational intervention with cognitive behavioral therapy components. The RCT consisted of a 10-week period during which the interventions were delivered, followed by a 26-week follow-up period. The recruitment began in August 2012 and ended in February 2013. The last follow-up measurement was performed in December 2013. The RCT was performed in 3 study centers in Finland—Jyväskylä, Kuopio, and Helsinki. The purpose of the lifestyle interventions was to improve the participants’ well-being and activate them to make beneficial changes in their everyday life. The design of the RCT was described in detail by Lappalainen et al [34]. See Multimedia Appendix 1 for the CONSORT-EHEALTH checklist [35].

The primary outcome of the RCT was psychological flexibility (measured with the Acceptance and Action Questionnaire II, AAQ-II [36]). The main analysis of the RCT found that although AAQ-II did not change significantly, psychological flexibility related to weight issues (Acceptance and Action Questionnaire for Weight-Related Difficulties, AAQW) improved significantly in both ACT intervention groups (in manuscript in preparation by Marjukka Kolehmainen and colleagues). This paper presents a secondary analysis, which focused solely on the mobile intervention group and investigated the usage of the mobile intervention and the dose-response relationship between usage and the change in psychological flexibility (AAQ-II).

### Participants

Participants were recruited through newspaper advertisements seeking overweight individuals suffering from psychological stress. The inclusion criteria were (1) age between 25 and 60 years, (2) body mass index (BMI) of 27-34.9 kg/m^2^, (3) psychological stress (at least 3/12 points in the General Health Questionnaire (GHQ-12 [37]), and (4) the possibility to use a computer and Internet connection. Out of the 645 individuals who responded to the advertisements, 339 fulfilled the inclusion criteria and consented to participate. After baseline examinations, further 41 participants declined to participate or were excluded because of findings in baseline measurements. Thus, 298 participants started the actual study. The participants consisted of 48 males and 250 females. Their mean age was 49.0 (SD 7.6) years and BMI was 31.1 (SD 3.0) kg/m^2^.

In this study, only the mobile intervention participants were analyzed. Altogether, 85 of 339 (25.1%) participants were randomized to the mobile intervention group. Of these, 78 (92%) received the intervention, and 75 (88%) participated in postintervention measurements. One participant did not provide postintervention results on psychological flexibility and thus was excluded, leaving 74 (87%) participants for the analyses.

### Mobile Intervention

**Figure 1:**
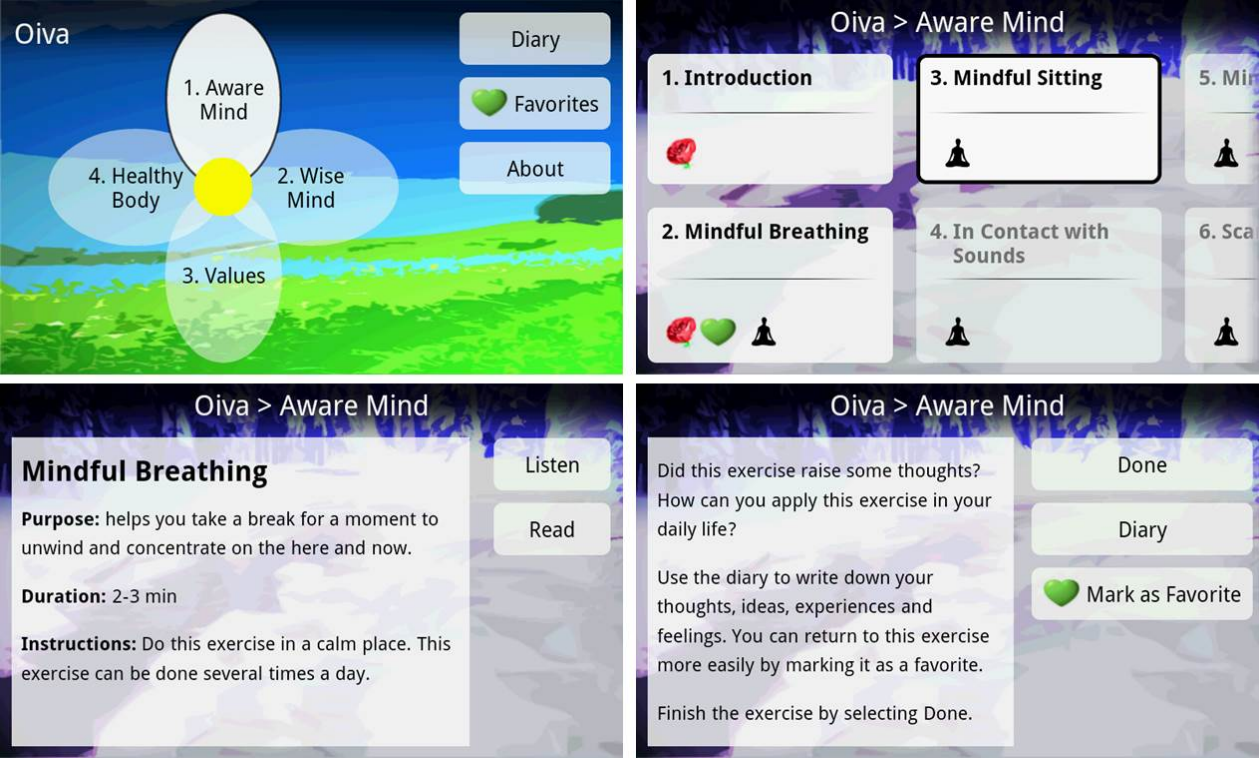
Screenshots of Oiva app: main view (top left), exercise browser (top right), instructions for an exercise (bottom left), and reflection screen (bottom right).

The mobile intervention group received their ACT intervention through a mobile application, called Oiva Figure 1, which has been previously described by Ahtinen et al. [37]. The aim of the app was to improve psychological flexibility by teaching the users the 6 core processes of ACT. The processes are (1) *Being present*, that is, having a nonjudgmental contact with psychological and environmental events; (2) *Self as context*, that is, being aware of one’s flow of experiences without attachment to or investment in them; (3) *Acceptance*, that is, actively embracing feelings and inner events without trying to change them; (4) *Cognitive defusion*, that is, changing the undesirable functions of thoughts and feelings, for example, by observing them without attachment to them; (5) *Values*, that is, identifying things that are truly important to an individual and that help determine life directions; and (6) *Committed action*, that is, committing to concrete goals and actions based on personal values [19].

The content was provided as 45 exercises, divided into 4 main modules. Each exercise was coded according to the ACT process or processes it addressed. There were 14 exercises related to the *Being present* process including, for example, a mindful sitting exercise and a mindful eating exercise [39]. The *Self as context* process had 5 exercises, for example, the “Floating leaves on a moving stream” metaphor [39]. The *Cognitive defusion* process had 9 exercises, for example, the “Passengers on a bus” metaphor [40]. The *Acceptance* process had 8 exercises, for example, the “Tug of war with a monster” metaphor [40]. The *Values* process had 8 exercises, for example, the “Attending your own funeral” exercise [39]. The *Committed action* process had 10 exercises, for example, a goal-setting exercise [39]. Of the exercises, 9 were associated with 2 processes.

Most exercises were short, taking about 1-3 minutes to complete, and were provided both in text and in audio format in Finnish. The participants were free to use the exercises in any order while the app provided subtle guidance by highlighting the next recommended exercise and module. The aim was to guide the users to proceed from easier exercises and basic skills to more challenging ones. A feasibility study was conducted to ensure the app’s readiness for the RCT, reported by Ahtinen et al [38]. Currently, mobile and Web versions of the app are available in Finnish [41-43].

In the beginning of the study, the participants were invited to a 1.5-hour group meeting where a trained psychologist gave a 30-minute presentation about the principles of ACT. The features of the mobile app were introduced by a researcher. The participants were given Android mobile phones (ZTE Blade or ZTE Skate; ZTE Corporation, Shenzhen, China) with the app preinstalled along with printed user instructions. The participants were allowed to use the phones as their personal phones, but as the intervention period was only 8 weeks, it was not expected of them. The phones and the app were briefly tested in the group to make sure everyone knew how to use them. The participants were encouraged to use the app independently and actively, a few times a week, for the following 8 weeks. The participants did not get any feedback or support during the course of the intervention. The suggested number of exercises to be performed per session was 1-3, but the participants were encouraged to find personally appropriate ways of use. At the end of the intervention period, the participants attended postintervention measurements and returned their phones. Researchers had no access to the app during the intervention, and the app and its contents remained constant during the intervention.

### Measurements

This study analyzed the data collected during the baseline measurements and postintervention measurements of the RCT as well as the usage log data collected by the mobile app. The psychological measurements and prior experience in using technologies were collected through Web-based questionnaires.

The main outcome measure of the study was psychological flexibility—or, more precisely, psychological inflexibility—measured with the AAQ-II [36]. The AAQ-II is a 7-item questionnaire that assesses experiential avoidance and psychological inflexibility. The statements of the questionnaire (eg, “I’m afraid of my feelings” or “Worries get in the way of my success”) are rated on a scale from 1 (=never true) to 7 (=always true). Higher scores on the AAQ-II reflect lower levels of psychological flexibility. Thus, improvement in psychological flexibility is defined as a decreased score on the AAQ-II.

Weight-specific psychological flexibility was measured using the AAQW [44], which is a 22-item questionnaire measuring acceptance of weight-related thoughts and feelings and their interference with valued actions. The statements include, for example, “I try hard to avoid feeling bad about my weight and how I look” and “If I’m overweight, I can’t live the life I want” and are rated on a scale from 1 (=never true) to 7 (=always true) [44]. Higher scores on the AAQW reflect lower psychological flexibility regarding weight.

Participants’ prior experience in using smart mobile phones, mobile wellness apps, and wellness devices was assessed in the beginning of the study as part of the baseline questionnaire. Each participant was assigned 3 binary attributes based on his or her prior experience in using technologies: (1) Smart mobile phone owner (if the participant owned a smart mobile phone before joining the study), (2) mobile wellness user (if the participant had used wellness-specific apps on the mobile phone), and (3) wellness device user (if the participant had used wellness devices, such as pedometers or heart rate monitors).

### Usage Metrics

The mobile app recorded usage log files locally in the mobile phone. The log files were obtained from the phones at the end of the intervention period. The log files were analyzed to extract altogether 15 usage metrics, detailed in Table 1. The usage metrics can be divided into 3 categories: metrics describing the intensity of use (metrics 1-7 in Table 1), metrics describing the usage of content (metrics 8-13), and metrics describing the ways of use (metrics 14 and 15).

First, individual usage sessions were identified and their durations were calculated. Then, individual exercises performed, and whether they were performed by reading or listening, were identified. The usage of exercises related to the 6 processes of ACT was studied based on the coding of exercises. First, the number of exercises performed was calculated for each process and normalized by the number of exercises belonging to the process, to account for there being a different number of exercises related to the processes (varying between 5 and 14). Then, a percentage of use compared with the total number of exercises performed by the participant was calculated for each process, indicating what the participants focused on the most.

**Table 1:** Usage metrics.

Usage metric	Description
Number of usage sessions	Total number of usage sessions performed by a participant.
Number of usage days	The number of days containing the start of a usage session.
Number of usage weeks	The number of weeks containing a usage session.
Number of exercises	The number of exercises performed by a participant, containing also repeated exercises.
Total duration of use	The sum of all sessions’ durations in hours.
Session duration	The mean duration of individual sessions in minutes.
Completion %	Percentage of all exercises performed, that is, program completion percentage.
Being present %	Percentage of exercises performed related to the “Being present” process.
Self as context %	Percentage of exercises performed related to the “Self as context” process.
Cognitive defusion %	Percentage of exercises performed related to the “Cognitive defusion” process.
Acceptance %	Percentage of exercises performed related to the “Acceptance” process.
Values %	Percentage of exercises performed related to the “Values” process.
Committed action %	Percentage of exercises performed related to the “Committed action” process.
Usage pattern	The ratio of sessions during the first half of the intervention period versus the second half.
Listen %	Percentage of exercises performed by listening.

### Analysis

The dose-response relationship between the usage metrics and the change in psychological flexibility was analyzed using linear regression analyses on continuous variables. First, univariate linear regression models were used, and then potential confounders (age, gender, baseline GHQ-12 score, and baseline AAQ-II and AAQW scores) were added to the models. Regression coefficients (B’s) with their 95% confidence intervals and significance levels were reported for both unadjusted and adjusted models. In addition, the *R*^2^ values were reported for the unadjusted models to assess the magnitude of the effect.

To assess the effects of usage between those whose psychological flexibility improved and those whose flexibility did not improve, binary logistic regression analyses were conducted. The participants were divided into 2 groups based on the change in psychological flexibility during the intervention. Those whose psychological flexibility increased (ie, the change in the AAQ-II score was negative) were labeled “improvers” and those whose psychological flexibility remained unchanged or decreased (ie, the change in the AAQ-II score was zero or positive) were labeled “nonimprovers.” The cutoff of zero was used because AAQ-II does not have reference values for clinically significant change. First, univariate logistic regression models were used to test the independent effect of each usage metric and then, multiple regressions were employed to adjust for the potential confounders. The odds ratios (ORs) with their 95% confidence intervals and significance values were reported both for the unadjusted and for adjusted models. In addition, medians and interquartile ranges (IQRs) of usage metrics were reported for both groups.

The usage metrics found to be significant in the regression analyses were further investigated for associations with baseline variables. As the distributions of the usage metrics were skewed, Spearman correlation was used for continuous variables and Mann-Whitney *U* test for cases where 1 variable was categorical.

The statistical analyses were performed using the IBM SPSS Statistics version 22 (IBM Corp, Armonk, NY, USA). Statistical significance was set at *P*<.05.

### Ethical Approval

The RCT was approved by the ethics committee of the Central Finland Health Care District, and written informed consent was obtained from all participants. The approval and consent also covered the secondary analyses performed. The trial was registered at ClinicalTrials.gov with the identifier NCT01738256.

## Results

### Mobile Intervention Participants

The mean level of psychological flexibility in the mobile ACT intervention group was 20.4 (95% CI 18.3-22.5) at baseline and 18.5 (95% CI 16.4-20.7) at postintervention. The mean change was −1.9 (95% CI −3.2 to −0.5) and the within-group effect size (Cohen’s *d*) was 0.2, indicating a small improvement in psychological flexibility.

Table 2 presents the participants’ baseline characteristics. The baseline score of psychological flexibility (AAQ-II) was significantly correlated with the change in psychological flexibility during the intervention (ρ=−.33, *P*=.004). Also, the correlation between weight-related psychological flexibility (AAQW) and the change in psychological flexibility indicated slight association (ρ=−.20, *P*=.09).

**Table 2:** Participants’ baseline characteristics: age, gender, body mass index, education, prior technology experiences, and psychological characteristics.

Characteristic	Participants, n=74
Age^a^in years, median (IQR^b^)	49.6 (45.4-55.3)
Gender, female, n (%)	64 (86)
BMI^c^(kg/m^2^), mean (SD)	31.5 (2.8)
Education (college or higher), n (%)	59 (80)
Smart mobile phone owner, n (%)	25 (34)
Mobile wellness user, n (%)	9 (12)
Wellness device user, n (%)	60 (81)
AAQ-II^d^, mean (SD)	20.4 (9.1)
AAQW^e^, mean (SD)	88.8 (21.0)
GHQ-12^a,f^, median (IQR)	6.0 (5.0-9.0)

^a^ Skewed distribution.

^b^ IQR: interquartile range.

^c^ BMI: body mass index.

^d^ AAQ-II: Acceptance and Action Questionnaire II.

^e^ AAQW: Acceptance and Action Questionnaire for Weight-Related Difficulties.

^f^ GHQ-12: 12-item General Health Questionnaire.

When the participants were divided into 2 groups (improvers, n=38; and nonimprovers, n=36), the mean change from baseline to postintervention in psychological flexibility was −6.3 (95% CI −7.6 to −5.0) for improvers and 2.8 (95% CI 1.6-3.9) for nonimprovers (*P*<.001). The within-group effect size was 0.8 for improvers and −0.3 for nonimprovers.

### Usage Metrics

The mobile app was available to the mobile intervention participants for a median of 58 days (IQR 55-60). During this period, they used the app as follows. The median number of usage sessions was 21 (IQR 12-35), the number of usage days was 15 (IQR 9.0-24), and the number of usage weeks was 7.0 (IQR 4.0-8.0). Less than half of the participants, 31/74 (42%), used the app for 8 weeks or more.

The median total duration of use was 4.7 (IQR 3.2-7.2) hours and the number of exercises performed was 63 (IQR 46-98). The median session duration was 13.5 (IQR 9.8-17) minutes. The median completion percentage was 91% (IQR 64%-96%), that is, the participants completed most exercises. The most used exercise types were *Being present* (26%, IQR 20%-33%) and *Self as context* (24%, IQR 17%-30%).

The usage was mostly focused on the first half of the intervention period, as the median usage pattern was 2.4, IQR 1.7-4.1. Most exercises (81%, IQR 63%-92%) were performed by listening instead of reading.

### Impact of App Use on Psychological Flexibility

#### Dose Response

Table 3 presents the results of the linear regression analyses.The strongest association with psychological flexibility in the unadjusted analyses was seen with the use of *Self as context* related exercises (B=0.19, *P*=.002, *R*^2^=.12), indicating that active usage of these exercises was associated with decreased psychological flexibility. The adjusted analyses revealed a dose-response relationship with the majority of metrics describing the intensity of use, indicating that a higher number of usage sessions (B=−0.10, *P*=.01), usage days (B=−0.17, *P*=.008), usage weeks (B=−0.73, *P*=.02), and exercises (B=−0.02, *P*=.03); the total duration of use (B=−0.30, *P*=.04); and the use of *Acceptance* related exercises (B=−0.18, *P*=.04) predicted increased psychological flexibility. These parameters were also borderline significant in the unadjusted models.

**Table 3:** Linear regression analyses between usage metrics and change in Acceptance and Action Questionnaire II score.

Usage metric	Β (95% CI)^a^	*R* ^2^	*P* ^a^	B (95% CI)^b^	*P* ^b^
Number of usage sessions	−0.07 (−0.15 to 0.01)	.05	.07	−0.10 (−0.18 to −0.02)	.01
Number of usage days	−0.12 (−0.24 to 0.01)	.05	.06	−0.17 (−0.30 to −0.05)	.008
Number of usage weeks	−0.61 (−1.22 to −0.01)	.05	.047	−0.73 (−1.34 to −0.11)	.02
Number of exercises	−0.02 (−0.04 to 0.003)	.04	.09	−0.02 (−0.05 to −0.002)	.03
Total duration of use, hours	−0.24 (−0.51 to 0.03)	.04	.08	−0.30 (−0.59 to −0.02)	.04
Session duration, minutes	0.01 (−0.19 to 0.22)	0	.91	0.05 (−0.15 to 0.26)	.60
Completion %	−0.004 (−0.06 to −0.05)	0	.87	−0.03 (−0.08 to 0.03)	.33
Being present %	−0.03 (−0.10 to 0.45)	.01	.43	−0.004 (−0.08 to 0.07)	.92
Self as context %	0.19 (0.07-0.31)	.12	.002	0.22 (0.10-0.34)	.001
Acceptance %	−0.13 (−0.31 to 0.04)	.03	.14	−0.18 (−0.36 to −0.01)	.04
Cognitive defusion %	−0.14 (−0.35 to 0.09)	.02	.23	−0.19 (−0.42 to 0.04)	.10
Values %	0.06 (−0.18 to 0.31)	0	.61	−0.04 (−0.31 to 0.22)	.75
Committed action %	−0.05 (−0.33 to 0.23)	0	.72	−0.16 (−0.46 to 0.13)	.28
Usage pattern	0.45 (−0.01 to 0.91)	.05	.06	0.35 (−0.12 to 0.82)	.14
Listen %	0.06 (−0.005 to 0.12)	.05	.07	0.05 (−0.01 to 0.11)	.12

^a^ Unadjusted.

^b^ Adjusted for baseline values of age, gender, 12-item General Health Questionnaire, Acceptance and Action Questionnaire II, and Acceptance and Action Questionnaire for Weight-Related Difficulties.

When all usage metrics found to be significant in the adjusted analyses were entered together into a linear regression model without adjustments, the adjusted *R*^2^ was .05

#### Binary Effect

Table 4 presents the results of the binary logistic regression analyses assessing the effects of usage between improvers and nonimprovers. Median (IQR) values of usage metrics in the improvers’ and nonimprovers’ groups were presented. The adjusted models identify similar parameters as found with linear regression models. The odds of improved psychological flexibility increased significantly along with the number of usage sessions (OR=1.08, *P*=.002), usage days (OR=1.13, *P*=.001), usage weeks (OR=1.48, *P*=.005), and exercises (OR=1.02, *P*=.003), and the total duration of use (OR=1.42, *P*=.002). Regarding the type of exercises used, the odds of improved psychological flexibility increased with active use of *Acceptance* exercises (OR=1.15, *P*=.005) and *Cognitive defusion* exercises (OR=1.15, *P*=.01) and decreased with active use of *Self as context* exercises (OR=0.90, *P*=.005). The results were similar also in the unadjusted models.

**Table 4:** Logistic regression analysis; associations between usage parameters and improvement in psychological flexibility.

Usage parameter	Improvers (n=38) median (IQR^a^)	Nonimprovers (n=36) median (IQR)	OR^b,c^ (95% CI)	*P* ^c^	OR^d^ (95% CI)	*P* ^d^
Number of usage sessions	27.5 (16.3-41.0)	18.0 (10.0-23.8)	1.05 (1.02-1.10)	.005	1.08 (1.03-1.13)	.002
Number of usage days	19.0 (13.0-28.3)	11.5 (8.0-16.0)	1.09 (1.03-1.15)	.004	1.13 (1.05-1.21)	.001
Number of usage weeks	8.0 (5.8-8.0)	5.5 (4.0-7.0)	1.38 (1.09-1.74)	.007	1.48 (1.13-1.95)	.005
Number of exercises	82.5 (51.8-148)	51.5 (39.5-79.0)	1.02 (1.00-1.03)	.009	1.02 (1.01-1.04)	.003
Total duration of use, hours	6.0 (3.7-11.1)	4.0 (2.6-6.0)	1.27 (1.08-1.51)	.005	1.42 (1.14-1.76)	.002
Session duration, minutes	13.5 (9.9-17.5)	13.7 (9.6-17.7)	0.99 (0.93-1.06)	.84	0.97 (0.90-1.05)	.48
Completion %	93 (86-98)	83 (56-93)	1.01 (0.99-1.03)	.17	1.02 (1.00-1.05)	.06
Being present %	21 (16-28)	28 (21-38)	0.99 (0.97-1.02)	.61	0.99 (0.96-1.01)	.33
Self as context %	21 (14-28)	26 (21-36)	0.93 (0.88-0.98)	.006	0.90 (0.84-0.97)	.005
Acceptance %	13 (11-20)	11 (5-14)	1.10 (1.03-1.19)	.009	1.15 (1.04-1.27)	.005
Cognitive defusion %	15 (11-19)	11 (8-15)	1.11 (1.02-1.20)	.02	1.15 (1.03-1.27)	.01
Values %	11 (9-14)	11 (8-14)	0.99 (0.91-1.07)	.74	1.01 (0.92-1.11)	.86
Committed action %	11 (7-13)	9 (6-11)	1.06 (0.97-1.17)	.20	1.11 (0.98-1.25)	.09
Usage pattern	2.1 (1.5-2.9)	3.1 (1.9-5.7)	0.88 (0.75-1.04)	.15	0.92 (0.78-1.10)	.37
Listen %	81 (55-91)	81 (70-92)	0.98 (0.96-1.01)	.19	0.99 (0.96-1.01)	.26

^a^ IQR: interquartile range.

^b^ OR: odds ratio.

^c^ Unadjusted.

^d^ Adjusted for baseline values of age, gender, 12-item General Health Questionnaire, Acceptance and Action Questionnaire II, and Acceptance and Action Questionnaire for Weight-Related Difficulties.

### Associations Between Usage Parameters and Baseline Variables

The usage metrics that were found to be significant predictors of changes in psychological flexibility (Tables 3 and 4) were analyzed for associations with the participants’ baseline characteristics (Table 2).

Older participants used the app more actively, as indicated by the number of exercises (ρ=.25, *P*=.03) and the total duration of use (ρ=.25, *P*=.04). Older participants used the *Self as context* exercises (ρ=−.33, *P*=.005) less than younger participants.

Women used the app more than men in terms of the number of usage sessions (median 23, IQR 13-38 vs median 14, IQR 8.8-19; *P*=.04), usage days (median 16, IQR 10-25 vs median 11, IQR 5.8-16; *P*=.03), and usage weeks (median 7, IQR 5.0-8.0 vs median 4, IQR 3.0-8.0; *P*=.04).

Participants who owned a smart mobile phone before the study were less likely to use the app actively than those who did not own a smart mobile phone, based on the number of usage days (median 11, IQR 6-18 vs median 16, IQR 12-25; *P*=.02) and usage weeks (median 5.0, IQR 3.0-8.0 vs median 7.0, IQR 5.5-8.0; *P*=.005), the number of exercises (median 52, IQR 25-71 vs median 79, IQR 50-108; *P*=.02), and the total duration of use (median 220 minutes, IQR 131-324 vs median 319 minutes, IQR 227-461; *P*=.01). Smart mobile phone owners also used *Cognitive defusion* exercises (median 11%, IQR 7%-16% vs median 14%, IQR 11%-18%; *P*=.04) and *Acceptance* exercises (median 11%, IQR 3%-13% vs median 13%, IQR 11%-19%; *P*=.003) less.

## Discussion

### Principal Findings

This study focused on metrics describing the usage of a mobile ACT intervention and their associations with the change in psychological flexibility during an 8-week intervention. The purpose of the ACT intervention was to increase psychological flexibility and thereby support value-based approach in life. The aims of this study were to investigate the dose-response relationship between usage and the change in psychological flexibility and to identify the usage metrics most strongly associated with improvement. The importance of psychological flexibility has been highlighted in a review by Kashdan and Rottenberg [45] describing flexibility as a fundamental aspect of health. There is ample evidence on the value of psychological flexibility. For example, in many forms of psychopathology, the processes associated with flexibility are absent. It is also proposed that psychological flexibility may be helpful not only to people suffering from different pathologies but also to highly functioning people in finding greater efficacy and fulfillment in their daily lives [45].

On the group level, the mean change in psychological flexibility was −1.9 points, indicating a small improvement with an effect size of 0.2. The psychological flexibility of about half of the participants (38/74, 51%) increased and the mean change in this group was −6.3, indicating a large improvement with an effect size of 0.8. In comparison, Ly et al [29] reported a within-group effect size of 0.5 for psychological flexibility for an ACT-based mobile self-help program.

A dose-response relationship between usage and the change in psychological flexibility was found. The usage metrics that were the most strongly associated with the change in psychological flexibility were related to the intensity of use, that is, the number of usage sessions, the number of usage days, the number of usage weeks, the number of exercises performed, and the total duration of use. Logistic regression analyses confirmed these findings. However, the *R*^2^ values of individual usage metrics, ranging from .04 to .12, as well as the *R*^2^ of the combined model (.05) consisting of all significant usage metrics showed that the predictive power of the usage metrics was relatively low. Interestingly, the session duration was not associated with improvement of psychological flexibility. This may be due to the fact that as the exercises were relatively short, the session duration was naturally rather limited.

A dose-response relationship was found also for 2 metrics describing content usage. Active usage of exercises related to the *Acceptance* process and less active usage of *Self as context* related exercises predicted increased psychological flexibility. Logistic regression analyses confirmed these findings and also indicated that more active usage of *Cognitive defusion* related exercises was associated with increased flexibility. Hayes et al [19] found that, based on a series of small-scale dismantling studies, *Acceptance* and *Cognitive defusion* related exercises had the strongest evidence of effectiveness.

When comparing participants whose psychological flexibility increased (improvers) and whose did not increase (nonimprovers), we saw that the total duration of use was more than 6 hours for improvers compared with 4 hours for nonimprovers. This result highlights that, even when an intervention is delivered through a mobile app, the participants need to commit substantial time and effort to the intervention in order to gain benefits. In comparison, in the study by Bricker et al [23] the average total usage time of the Web-based smoking cessation intervention was about 2.9 hours, and in the study by Carlbring et al [24] a depression intervention was used, on average, for 3.5 hours.

Older age, female gender, and not owning a smart mobile phone before the study were associated with more active use. The reason why not owning a smart mobile phone before the study affected usage may be that participants who already had a smart mobile phone were more reluctant to take a secondary phone into use. Another factor may be that those who did not own a smart mobile phone before were actually motivated by receiving a new phone for a few weeks.

### Comparison With Prior Work

Donkin et al [11] investigated the associations between several usage metrics and improvement in depression during a 12-week Internet intervention. Similar metrics and associations with outcome as in our study were found for the following usage metrics: (1) the total number of minutes spent in the program and (2) the total number of activities completed. There was also an interesting similarity in the total usage time in these 2 studies, despite the fact that one of the interventions was delivered through the Internet whereas the other was a mobile app. Donkin et al [11] reported an average total usage time of 5.9 hours for those whose depression improved compared with 4.9 hours for those who did not achieve improvements. In our study, the median total usage time was 6.0 hours for improvers versus 4.0 hours for nonimprovers. Ly et al [32] reported a study of an ACT-based mobile intervention for stress management. In their study, 16/36 (44%) participants adhered to the study for all of the 6 weeks of the intervention. In our study, using a similar criterion, 31/74 (42%) participants used the mobile app weekly for the entire 8-week intervention period.

Studying the usage of content in digital interventions is a relatively new area of research, made possible by automatic and detailed tracking of usage. Only a few studies have attempted to investigate the associations between the usage of content and improvement in mobile interventions. Heffner et al [33] studied the usage of a mobile ACT intervention for smoking cessation and found that only 2 of the 10 most popular features were associated with smoking abstinence and, furthermore, that there were several features among the less popular ones that were associated with smoking abstinence. Our study provided preliminary evidence that focusing on *Acceptance* and *Cognitive defusion* related exercises was associated with improvement of psychological flexibility, whereas focusing on *Self as context* related exercises actually decreased the likelihood of improvement. These results suggest that *Acceptance* and *Cognitive defusion* related exercises are needed in addition to *Self as context* related exercises in order to gain a significant effect on flexibility. Identifying the most effective components of interventions is important because it enables better design of interventions and making sure that users are exposed to those components instead of or in addition to the less effective ones. For example, in our app, most *Self as context* related exercises were presented earlier than *Acceptance* and *Cognitive defusion* related exercises. As many participants proceeded in the app in the suggested order, those who used the app less may not have been exposed to the more advanced exercises appearing later in the app as extensively.

Many studies have tried to identify demographic predictors of usage and depending on the type of intervention, different and often conflicting predictors have been found. Female gender has often predicted better adherence [8,46]. This was the case also in our study, but as there were very few men involved in the study, strong conclusions cannot be drawn from this result. Sometimes older [8,17,47] and sometimes younger age [8] have been found to predict better adherence. In our study, older participants were more active users. Owning a smart mobile phone before the study was associated with less active usage, which is in line with the finding by Christensen et al [8] who found that participants who were less experienced with using computers adhered better. Also, lower symptom levels have been found to predict adherence, especially in psychological disorders [8]. In our study, no such associations were found.

Other important predictors of adherence to interventions reported in the literature include, for example, outcome expectancy, social support, and autonomous motivation [3,48,49]. The importance of these factors probably increases in the case of self-help and digital interventions where users need to find the time and initiative to use the intervention on their own. Although we did not measure motivation, we found that the baseline level of psychological flexibility correlated with the changes in flexibility, that is, those with a lower baseline level of psychological flexibility improved more. This may mean that those who gained more benefits may have had more room for improvement and may have felt a greater need—and motivation—to use the intervention. Also, RCT as the study setup and having counselor contact have been found to predict usage [9,15,16]. Although in our study the study procedures included only 1 face-to-face meeting and only a short presentation by a trained psychologist, it may have contributed to active usage along with all the study procedures related to the RCT setup.

### Limitations

The study population consisted of participants who volunteered to take part in the RCT. Although they were randomly allocated to different interventions and therefore did not know which intervention, if any, they would receive, the group is likely to be biased and the results cannot be generalized. Volunteering in a research study often requires a lot of effort from the participants and they may therefore be highly motivated. Also, the large proportion of female participants, which is not uncommon in health trials, limits making generalizations.

The baseline level of psychological flexibility correlated significantly with the change in psychological flexibility. This means that those who had more room for improvement also experienced larger effects. It is not possible to evaluate whether this is due to higher motivation, greater perceived need, or features of the app.

We acknowledge that we performed a large number of statistical tests without correcting the statistical significance level of .05, which means that some of the low *P* values may have occurred by chance. However, this study was exploratory by nature and any results should be confirmed in future studies.

Regarding the design of the RCT, the duration of the mobile intervention (8 weeks) was arbitrary and does not correspond to real-life use of mobile apps. Because the participants had to return the phones at the end of the intervention period, there is no way of knowing how the usage would have evolved over time and whether additional benefits would have been gained as a result. On the other hand, the knowledge of the limited availability may also have motivated the participants for such active use. Also, the fact that the app did not run on the participants’ personal mobile phones probably affected the ways they used the app.

There were also some limitations related to the mobile app logs that were recorded automatically by the mobile phone. As the app did not require logging in and out for session, it could be running constantly in the background without being used, or the app could be closed accidentally during a usage session, which made the log files challenging to analyze. Therefore, the usage metrics may not be absolutely accurate. However, the rules for determining the usage metrics were verified by manually inspecting several random log files and therefore we can be fairly confident that the metrics describe actual usage satisfactorily.

### Conclusions and Future Research

The results indicated that active usage of a mobile ACT intervention was associated with improved psychological flexibility. Usage metrics related to the intensity of use and the usage of content were found to be the strongest predictors of the change in psychological flexibility. This study showed that rather intensive usage was required in order to gain benefits, and therefore the study highlights the need to measure and optimize the intensity of usage in mobile interventions. The study also implies that, to ensure effectiveness, the components known to be the most effective should be prioritized to make sure all users are exposed to them.

Future research should strive to study the usage of mobile apps in a more natural way, including using the participants’ personal mobile phones and not limiting usage time. The results provided in this study about the associations between usage of different types of exercises and outcomes highlight the capabilities of digital interventions to enable detailed analyses of what the participants are actually exposed to during interventions.

## Appendix

Multimedia Appendix 1 CONSORT-eHealth (V 1.6.1) checklist [35].

## Abbreviations

AAQ-II: Acceptance and Action Questionnaire II
AAQW: Acceptance and Action Questionnaire for Weight-Related Difficulties
ACT: Acceptance and Commitment Therapy
BMI: body mass index
GHQ-12: 12-item General Health Questionnaire
IQR: interquartile range
OR: odds ratio
RCT: randomized controlled trial

## References

[1] Sacks FM, Bray GA, Carey VJ, Smith SR, Ryan DH, Anton SD, McManus K, Champagne CM, Bishop LM, Laranjo N, Leboff MS, Rood JC, de Jonge L, Greenway FL, Loria CM, Obarzanek E, Williamson DA (2009). Comparison of weight-loss diets with different compositions of fat, protein, and carbohydrates. N Engl J Med.

[2] Patrick K, Calfas KJ, Norman GJ, Rosenberg D, Zabinski MF, Sallis JF, Rock CL, Dillon LW (2011). Outcomes of a 12-month web-based intervention for overweight and obese men. Ann Behav Med.

[3] Webber KH, Tate DF, Bowling JM (2008). A randomized comparison of two motivationally enhanced Internet behavioral weight loss programs. Behav Res Ther.

[4] Lewis B, Williams D, Dunsiger S, Sciamanna C, Whiteley J, Napolitano M, Bock B, Jakicic J, Getz M, Marcus B (2008). User attitudes towards physical activity websites in a randomized controlled trial. Prev Med.

[5] Powell J, Hamborg T, Stallard N, Burls A, McSorley J, Bennett K, Griffiths KM, Christensen H (2013). Effectiveness of a web-based cognitive-behavioral tool to improve mental well-being in the general population: randomized controlled trial. J Med Internet Res.

[6] Cavanagh K, Strauss C, Cicconi F, Griffiths N, Wyper A, Jones F (2013). A randomised controlled trial of a brief online mindfulness-based intervention. Behav Res Ther.

[7] Sabaté E (2003). Adherence to long-term therapies: evidence for action.

[8] Christensen H, Griffiths KM, Farrer L (2009). Adherence in internet interventions for anxiety and depression. J Med Internet Res.

[9] Kelders SM, Kok RN, Ossebaard HC, van Gemert-Pijnen JE (2012). Persuasive system design does matter: a systematic review of adherence to web-based interventions. J Med Internet Res.

[10] Donkin L, Christensen H, Naismith SL, Neal B, Hickie IB, Glozier N (2011). A systematic review of the impact of adherence on the effectiveness of e-therapies. J Med Internet Res.

[11] Donkin L, Hickie IB, Christensen H, Naismith SL, Neal B, Cockayne NL, Glozier N (2013). Rethinking the dose-response relationship between usage and outcome in an online intervention for depression: randomized controlled trial. J Med Internet Res.

[12] Wildeboer G, Kelders SM, van Gemert-Pijnen JE (2016). The relationship between persuasive technology principles, adherence and effect of web-Based interventions for mental health: A meta-analysis. Int J Med Inform.

[13] Andersson G, Cuijpers P (2009). Internet-based and other computerized psychological treatments for adult depression: a meta-analysis. Cogn Behav Ther.

[14] Andrews G, Cuijpers P, Craske MG, McEvoy P, Titov N (2010). Computer therapy for the anxiety and depressive disorders is effective, acceptable and practical health care: a meta-analysis. PLoS One.

[15] Bennett-Levy J, Richards D, Farrand P, Christensen H, Griffiths K, Kavanagh Dj, Klein B, Lau M, Proudfoot J (2010). Turn on, tune in and (don´t) drop out: Engagement, adherence, attrition, and alliance with internet-based interventions. Oxford guide to low intensity CBT interventions.

[16] Titov N, Andrews G, Choi I, Schwencke G, Johnston L (2009). Randomized controlled trial of web-based treatment of social phobia without clinician guidance. Aust NZ J Psychiatry.

[17] Mattila E, Orsama A, Ahtinen A, Hopsu L, Leino T, Korhonen I (2013). Personal health technologies in employee health promotion: usage activity, usefulness, and health-related outcomes in a 1-year randomized controlled trial. JMIR Mhealth Uhealth.

[18] Hedman E, Carlbring P, Ljótsson B, Andersson G (2014). Internetbaserad psykologisk behandling: Evidens, indikation och praktiskt genomförande.

[19] Hayes SC, Luoma JB, Bond FW, Masuda A, Lillis J (2006). Acceptance and commitment therapy: model, processes and outcomes. Behav Res Ther.

[20] Bond FW, Bunce D (2003). The role of acceptance and job control in mental health, job satisfaction, and work performance. J Appl Psychol.

[21] Buhrman M, Skoglund A, Husell J, Bergström K, Gordh T, Hursti T, Bendelin N, Furmark T, Andersson G (2013). Guided internet-delivered acceptance and commitment therapy for chronic pain patients: a randomized controlled trial. Behav Res Ther.

[22] Hesser H, Gustafsson T, Lundén C, Henrikson O, Fattahi K, Johnsson E, Zetterqvist WV, Carlbring P, Mäki-Torkko E, Kaldo V, Andersson G (2012). A randomized controlled trial of Internet-delivered cognitive behavior therapy and acceptance and commitment therapy in the treatment of tinnitus. J Consult Clin Psychol.

[23] Bricker J, Wyszynski C, Comstock B, Heffner JL (2013). Pilot randomized controlled trial of web-based acceptance and commitment therapy for smoking cessation. Nicotine Tob Res.

[24] Carlbring P, Hägglund M, Luthström A, Dahlin M, Kadowaki Å, Vernmark K, Andersson G (2013). Internet-based behavioral activation and acceptance-based treatment for depression: a randomized controlled trial. J Affect Disord.

[25] Lappalainen P, Langrial S, Oinas-Kukkonen H, Tolvanen A, Lappalainen R (2015). Web-Based Acceptance and Commitment Therapy for Depressive Symptoms With Minimal Support: A Randomized Controlled Trial. Behav Modif.

[26] Levin ME, Pistorello J, Seeley JR, Hayes SC (2014). Feasibility of a prototype web-based acceptance and commitment therapy prevention program for college students. J Am Coll Health.

[27] Räsänen P, Lappalainen P, Muotka J, Tolvanen A, Lappalainen R (2016). An online guided ACT intervention for enhancing the psychological wellbeing of university students: A randomized controlled clinical trial. Behav Res Ther.

[28] Bricker JB, Mull KE, Kientz JA, Vilardaga R, Mercer LD, Akioka KJ, Heffner JL (2014). Randomized, controlled pilot trial of a smartphone app for smoking cessation using acceptance and commitment therapy. Drug Alcohol Depend.

[29] Ly KH, Dahl J, Carlbring P, Andersson G (2012). Development and initial evaluation of a smartphone application based on acceptance and commitment therapy. Springerplus.

[30] Plaza I, Demarzo MMP, Herrera-Mercadal P, García-Campayo J (2013). Mindfulness-based mobile applications: literature review and analysis of current features. JMIR Mhealth Uhealth.

[31] Mani M, Kavanagh DJ, Hides L, Stoyanov SR (2015). Review and Evaluation of Mindfulness-Based iPhone Apps. JMIR Mhealth Uhealth.

[32] Ly KH, Asplund K, Andersson G (2014). Stress management for middle managers via an acceptance and commitment-based smartphone application: A randomized controlled trial. Internet Interventions.

[33] Heffner JL, Vilardaga R, Mercer LD, Kientz JA, Bricker JB (2015). Feature-level analysis of a novel smartphone application for smoking cessation. Am J Drug Alcohol Abuse.

[34] Lappalainen R, Sairanen E, Järvelä E, Rantala S, Korpela R, Puttonen S, Kujala UM, Myllymäki T, Peuhkuri K, Mattila E, Kaipainen K, Ahtinen A, Karhunen L, Pihlajamäki J, Järnefelt H, Laitinen J, Kutinlahti E, Saarelma O, Ermes M, Kolehmainen M (2014). The effectiveness and applicability of different lifestyle interventions for enhancing wellbeing: the study design for a randomized controlled trial for persons with metabolic syndrome risk factors and psychological distress. BMC Public Health.

[35] Eysenbach G, Consort-EHEALTH Group (2011). CONSORT-EHEALTH: improving and standardizing evaluation reports of Web-based and mobile health interventions. J Med Internet Res.

[36] Bond FW, Hayes SC, Baer RA, Carpenter KM, Guenole N, Orcutt HK, Waltz T, Zettle RD (2011). Preliminary psychometric properties of the Acceptance and Action Questionnaire-II: a revised measure of psychological inflexibility and experiential avoidance. Behav Ther.

[37] Makowska Z, Merecz D, Mościcka A, Kolasa W (2002). The validity of general health questionnaires, GHQ-12 and GHQ-28, in mental health studies of working people. Int J Occup Med Environ Health.

[38] Ahtinen A, Mattila E, Välkkynen P, Kaipainen K, Vanhala T, Ermes M, Sairanen E, Myllymäki T, Lappalainen R (2013). Mobile mental wellness training for stress management: feasibility and design implications based on a one-month field study. JMIR Mhealth Uhealth.

[39] Hayes S, Smith S (2005). Get out of your mind and into your life: The new acceptance and commitment therapy.

[40] Hayes Sc, Strosahl Kd, Wilson Kg (1999). Acceptance and commitment therapy: An experiential approach to behavior change.

[41] Oiva on Google Play.

[42] Oiva on App Store.

[43] Web version of Oiva.

[44] Lillis J, Hayes SC (2008). Measuring avoidance and inflexibility in weight related problems. International Journal of Behavioral Consultation and Therapy.

[45] Kashdan TB, Rottenberg J (2010). Psychological flexibility as a fundamental aspect of health. Clin Psychol Rev.

[46] Robroek SJ, van Lenthe FJ, van Empelen P, Burdorf A (2009). Determinants of participation in worksite health promotion programmes: a systematic review. Int J Behav Nutr Phys Act.

[47] Neve MJ, Collins CE, Morgan PJ (2010). Dropout, nonusage attrition, and pretreatment predictors of nonusage attrition in a commercial Web-based weight loss program. J Med Internet Res.

[48] Duda J, Smart A, Tappe M (1989). Predictors of adherence in the rehabilitation of athletic injuries: An application of personal investment theory. J Sport Exerc Psychol.

[49] Geraghty AW, Wood AM, Hyland ME (2010). Attrition from self-directed interventions: investigating the relationship between psychological predictors, intervention content and dropout from a body dissatisfaction intervention. Soc Sci Med.

